# The delivery modes and morbidity/mortality in very preterm infants with birth weight <1,500 g: a retrospective cohort study

**DOI:** 10.3389/fpubh.2025.1671581

**Published:** 2025-10-13

**Authors:** Haoran Wang, Xiaoming Xu, Meng Zhang, Yang He

**Affiliations:** ^1^West China Second University Hospital, Sichuan University, Chengdu, China; ^2^Key Laboratory of Birth Defects and Related Diseases of Women and Children, Ministry of Education, Sichuan University, Chengdu, Sichuan, China; ^3^Department of Neonatology, West China Second University Hospital, Sichuan University, Chengdu, China

**Keywords:** very preterm infants, vaginal delivery, cesarean delivery, mortality, morbidity

## Abstract

**Introduction:**

Compare outcomes of very preterm infants (VPIs) with a birth weight<1,500 g based on delivery mode and propensity score matching (PSM).

**Methods:**

This was a retrospective study (2016–2021) of 1,375 VPIs (692 vaginal and 683 cesarean deliveries). PSM created 390 matched pairs. Outcomes included respiratory and neurological morbidity and mortality.

**Results:**

PSM revealed no significant difference between the two groups at baseline or after antenatal corticosteroids administration. The incidence rates of birth asphyxia, delivery room resuscitation, neonatal respiratory distress syndrome, use of pulmonary surfactants, pulmonary hemorrhage, and use of invasive ventilation in the vaginal delivery group after PSM were 42.3, 68.2, 65.9, 59.2, 7.2, and 36.7%, respectively. These rates were significantly lower than those in the cesarean delivery group (52.3, 83.3, 79.5, 70.8, 14.4, and 51.8%, respectively) (*p* < 0.05). The difference was more significant in infants with a gestational age of 28–31^+6^ weeks (*p* < 0.05). There were no significant differences in the incidence rates of intraventricular hemorrhage (IVH), severe IVH, periventricular leukomalacia, bronchopulmonary dysplasia, necrotizing enterocolitis (NEC), NEC surgery, premature infant retinopathy (ROP), severe ROP, late-onset neonatal sepsis, or mortality between the two groups. The mortality rate within 3 days in the vaginal delivery group was 7.7%, which was higher than that in the cesarean delivery group (3.3%), primarily in infants with a gestational age < 28 weeks (*p* < 0.05).

**Conclusion:**

Cesarean section reduced early mortality in VPIs <28 weeks but increased respiratory morbidity at 28–31^+6^ weeks, with no impact on other outcomes. Since 52.1% of the cesarean sections were emergency procedures, this may have biased the results.

## 1 Introduction

According to estimates by the World Health Organization (WHO), over 13.4 million babies were born prematurely in 2020, with at least 1 in 10 births classified as preterm (1). Preterm birth is more prevalent in developing countries and the number is gradually increasing (2). Prematurity may arise from natural childbirth or medical indications that necessitate preterm induction or cesarean delivery. Premature infants, particularly those born very preterm, are at a high risk for infections and mortality. Surviving infants often encounter long-term adverse outcomes, including developmental delays and reduced IQ (3), which significantly affect the survival rates and quality of life of very preterm infants (VPIs).

With the development of medical conditions, the rate of cesarean deliveries has increased. According to the WHO, the global cesarean delivery rate has steadily increased from approximately 7% in 1990 to 21% in 2021, with projections suggesting that may reach 29% by 2030 (4). Although cesarean delivery is a critical life-saving procedure, the short-term and long-term health risks to newborns are unknown. Various delivery methods have generated controversies regarding the prognosis of VPIs. A 2013 Cochrane systematic review found no significant differences in neonatal mortality rates, hypoxic–ischemic encephalopathy, birth asphyxia, neonatal respiratory distress syndrome (NRDS), or other complications between the two delivery modes (5). Recent studies have suggested that cesarean delivery may reduce mortality rates and the risk of intraventricular hemorrhage (IVH) in VPIs (6–8). However, the findings also indicated no significant differences in the incidence of IVH, necrotizing enterocolitis (NEC), and bronchopulmonary dysplasia (BPD) between infants delivered by the two modes (6). Nonetheless, most studies had various confounding factors, such as birth weight and gestational age, which may have influenced the accuracy of these results. This study retrospectively analyzed the clinical characteristics of VPIs with birth weights <1,500 g who were admitted to the Neonatology Department of our hospital. We compared the morbidity and mortality rates associated with different delivery modes using propensity score matching (PSM) to mitigate potential confounding factors. In this study, we matched covariates including gestational age, birth weight, early-onset neonatal sepsis (EOS), inborn characteristics, and multiple births. Our aim was to explore the impact of delivery mode on the morbidity and mortality of VPIs, providing a reference for the treatment direction of VPIs with different delivery methods.

## 2 Materials and methods

### 2.1 Ethics

This study was approved by the Ethics Committee of West China Second University Hospital, Sichuan University (2021011). The informed consent was waived in this trial.

### 2.2 Objectives

A retrospective cohort study was conducted on VPIs with birth weight <1,500 g admitted to West China Second University Hospital from January 2016 to December 2021.

Inclusion criteria: infants born at gestational age <32 weeks and birth weight <1,500 g.

Exclusion criteria: Gestational age and birth weight did not meet inclusion criteria; birth weight <1,500 g and/or gestational age <32 weeks but admission weight >1,500 g and/or corrected gestational age ≥32 weeks; and severe congenital malformations, such as heart malformations, gastrointestinal malformations.

### 2.3 Data collection

All clinical data were collected from the electronic medical record system of VPIs that met the inclusion criteria, including gestational age, birth weight, gender, mode of delivery, multiple births, care practice, and morbidities, such as birth asphyxia, NRDS, BPD, pulmonary hemorrhage (PH), IVH, periventricular leukomalacia (PVL), premature infant retinopathy (ROP), NEC, late-onset neonatal sepsis (LOS), and mortality.

### 2.4 Grouping

Vaginal delivery and cesarean delivery according to the delivery modes.

### 2.5 Standards and definitions

Complete course of schedule of administration of antenatal corticosteroid (ACS):≥4 doses ACS.

*NRDS*: Newborn infants develop progressive respiratory distress and cyanosis within a few hours after birth, leading to respiratory failure. Chest X-ray shows decreased translucency in both lung fields, with a ground-glass appearance or white lung, and may show signs of bronchial inflation.

*BPD*: oxygen dependence still exists when correcting the gestational age to 36 weeks of preterm.

*PH*: Massive pulmonary hemorrhage involving at least 2 lung lobes. Manifested as sudden onset of dyspnea or irregular respiration, with bleeding from the mouth, nose, pharynx, or endotracheal tube. Chest X-ray shows significantly increased density at both lung hilums.

*IVH*: The diagnosis is mainly performed by Doppler ultrasound, and classified according to the definition by Volpe JJ et al. in 2008 (9). Severe IVH is defined as grade III and above IVH.

*PVL*: Diagnosis through head MRI.

*ROP*: According to the international classification of ROP, ROP fundus lesions are divided into zones and stages (10). Severe ROP is defined as stage 3 and/or type 1.

*NEC*: Defined as ≥2 stages according to Bell standard (11).

*LOS*: sepsis confirmed clinically or by culture after 3 days of birth.

*Birth asphyxia*: mild birth asphyxia is defined as an Apgar score of ≤ 7 at 1 min or 5 min, accompanied by an umbilical arterial blood gas pH of < 7.2. Severe birth asphyxia is defined as an Apgar score of ≤ 3 at 1 min or ≤ 5 at 5 min, accompanied by an umbilical arterial blood gas pH of < 7. Neonatal asphyxia in this study included both mild and severe cases.

*Mortality*: Defined as the endpoint outcome measure.

### 2.6 Statistical methods

By applying a logistic regression model to calculate propensity scores, the covariates included gestational age, birth weight, EOS, inborn characteristics, and multiple births. Due to the lack of records on the administration of antenatal corticosteroids (ACS) by pregnant women in some cases, this factor was not included as a covariates. Only comparisons were made between the two groups in patients with ACS administration records. Nearest-neighbor matching was conducted within a caliper width of 0.02. Infants in the vaginal and cesarean delivery groups were matched in a 1:1 ratio. After applying PSM to the VPIs, the groups were matched based on their propensity scores. Propensity scores were computed using the R software version 4.4.1 (R Core Team, Vienna, Austria).

For the description of population characteristics, continuous variables that followed a normal distribution were presented as mean ± standard deviation, while those that did not follow a normal distribution were described as median [M (Q25, Q75)]. Categorical variables were reported as counts and percentages. For continuous variables, either a t-test or Mann–Whitney U test was selected based on the normality of the variable, while for categorical variables, either Pearson’s chi-square test or Fisher’s exact test was used for data analysis, depending on the total sample size and theoretical frequency. The data obtained in this study were analyzed using SPSS version 22.0 statistical software, with a *p* < 0.05 considered statistically significant.

## 3 Results

### 3.1 Baseline variables

From January 2016 to December 2021, 1,375 VPIs were included in the study, of which 692 were delivered vaginally and 683 via cesarean section, with a cesarean delivery rate of 49.7%. After PSM, 780 infants were included, with 390 infants in each group. Among cesarean section, there were 203 cases of emergency cesarean section and 187 cases of elective cesarean section. The demographic and clinical characteristics of the patients are summarized in Table 1. Before PSM, vaginally delivered VPIs showed lower birth weights, gestational ages, rates of inborn characteristics, and multiple births compared with those delivered via cesarean section (*p* < 0.05). Conversely, the rates of infants with birth weight of <1,000 g, gestational age of <28 weeks, and EOS were higher in the cesarean delivery group than those in the vaginal delivery group (*p* < 0.05). After PSM, the differences between the two groups disappeared. There was no significant difference in the gender distribution between the two groups before and after PSM. Furthermore, there was a significant difference in the number of VPIs with ACS administration between the two groups before PSM, which disappeared after PSM (Table 1). After PSM, 10 VPIs had abnormal fetal positions: nine cases were breech presentations (2 were delivered vaginally and seven via cesarean section) and one case was a shoulder presentation.

**Table 1:** Baseline characteristics of VPIs between groups before and after PSM.

PSM		Before PSM					After PSM				
		Vaginal delivery (692)	Cesarean delivery (683)	Total (1375)	χ^2^/Z	*p*	Vaginal delivery (390)	Cesarean delivery (390)	Total (780)	χ^2^/Z	*p*
BW		1,130 (930, 1,280)	1,240 (1076.25, 1,370)	1,180 (1,000,1,330)	−8.256	<0.001	1,220 (1057.5, 1,350)	1,200 (1,060, 1,350)	1,220 (1,060, 1,350)	−0.115	0.908
<1,000 g		227 (32.8)	113 (16.5)	340 (24.7)	52.784	<0.001	74 (19.0)	71 (18.2)	145 (18.6)	0.076	0.782
1,000 ~ 1,499 g		456 (65.9)	579 (84.8)	1,035 (75.3)			316 (81.0)	319 (81.8)	635 (81.4)		
GA		28.2 (27.0, 29.4)	30.0 (28.6, 30.6)	29.1 (28.0, 30.2)	−16.885	<0.001	29.2 (28.2, 30.0)	29.1 (28.2, 30.0)	29.2 (28.2, 30.0)	−0.289	0.773
<28w		280 (40.5)	63 (9.2)	343 (24.9)	186.727	<0.001	73 (18.7)	61 (15.6)	134 (17.2)	1.298	0.255
28 ~ 31^+6^w		403 (58.2)	629 (92.1)	1,032 (75.1)			317 (81.3)	329 (84.4)	646 (82.8)		
Inborn		572 (82.7)	620 (90.8)	1,192 (86.7)	10.186	0.001	334 (85.6)	332 (85.1)	666 (85.4)	0.041	0.839
Multiple		312 (45.1)	384 (56.2)	696 (50.6)	13.235	<0.001	185 (47.4)	187 (47.9)	372 (47.7)	0.021	0.886
EOS		108 (15.6)	70 (10.2)	178 (12.9)	9.899	0.002	47 (12.1)	53 (13.6)	100 (12.8)	0.413	0.520
Gender (male)		350 (50.6)	362 (53.0)	712 (51.8)	0.809	0.369	200 (51.3)	207 (53.1)	407 (52.2)	0.252	0.616
ACS (*n* = 1,220)*/(*n* = 691)#	No ACS	51 (8.2)	78 (13.1)	129 (10.6)	10.309	0.006	33 (9.8)	32 (9.1)	65 (9.4)	2.536	0.281
	1–3 doses ACS	199 (31.9)	204 (34.2)	403 (33.0)			118 (34.9)	105 (29.7)	223 (32.3)		
	≥4 doses ACS	374 (59.9)	314 (52.7)	688 (56.4)			187 (55.3)	216 (61.2)	403 (58.3)		

ACS (*n* = 1,220)*: before PSM, the administration of ACS in 155 VPIs were unknown, the remaining 1,220 VPIs were analyzed, among them, 624 were in vaginal delivery group, 596 were in cesarean delivery group. ACS (*n* = 691)#: after PSM, the administration of ACS in 89 VPIs were unknown, the remaining 691 VPIs were analyzed, among them, 338 were in vaginal delivery group,353 were in cesarean delivery group. Data are presented as *n* (%) and median (interquartile range). PSM, propensity score matching; BW, birth weight; GA, gestational age; EOS, early-onset neonatal sepsis.

### 3.2 Morbidities analysis

The incidence rates of birth asphyxia, NRDS, and PH in the vaginal delivery group after PSM were 42.3, 65.9, and 7.2%, respectively, which were significantly lower than those in the cesarean delivery group (52.3, 79.5, and 14.4%, respectively; *p* < 0.05). The rate of resuscitation in the delivery room was higher in the cesarean delivery group than that in the vaginal delivery group (72.8% vs. 90.3%, *p* < 0.05). The pulmonary surfactant (PS) utilization rate of in the vaginal delivery group was significantly lower than that in the cesarean delivery group (59.2% vs. 70.8%; *p* < 0.05). Furthermore, the invasive ventilation utilization rate in the vaginal delivery group was 36.7% compared with the 51.8% in the cesarean delivery group (*p* < 0.05). The mortality rate within 3 days in the vaginal delivery group was 7.4%, which was higher than that in the cesarean delivery group (3.3%, *p* < 0.05). There were no statistically significant differences between the two groups in the incidence rates of IVH, severe IVH, PVL, BPD, NEC, NEC surgery, ROP, severe ROP, LOS, or non-invasive ventilation support (Table 2).

**Table 2:** The morbidities of VPIs between groups after PSM (%).

Morbidities	Vaginal delivery (390)	Cesarean delivery (390)	Total (780)	χ^2^/Z	*p*
Birth asphyxia	165 (42.3)	204 (52.3)	369 (47.3)	7.823	0.005
Delivery room resuscitation	266 (68.2)	325 (83.3)	591 (75.8)	24.308	<0.001
NRDS	257 (65.9)	310 (79.5)	567 (72.7)	18.142	<0.001
PS	231 (59.2)	276 (70.8)	507 (65.0)	11.412	<0.001
PH	28 (7.2)	56 (14.4)	84 (10.8)	10.46	0.001
invasive ventilation	143 (36.7)	202 (51.8)	345 (44.2)	18.092	<0.001
IVH	126 (32.3)	120 (30.8)	246 (31.5)	0.214	0.644
Severe IVH	30 (7.7)	38 (9.7)	68 (8.7)	1.031	0.310
PVL	8 (2.1)	13 (3.3)	21 (2.7)	1.223	0.269
BPD	122 (31.3)	119 (30.5)	241 (30.9)	0.054	0.816
NEC	23 (5.9)	25 (6.4)	48 (6.2)	0.089	0.766
NEC surgery	8 (2.1)	10 (2.6)	18 (2.3)	0.227	0.633
ROP	121 (31.0)	122 (31.3)	243 (31.2)	0.006	0.938
Severe ROP	5 (1.3)	4 (1.0)	9 (1.2)	–	1.000
LOS	37 (9.5)	33 (8.5)	70 (9.0)	0.251	0.616
non-invasive ventilation	357 (91.5)	368 (94.4)	725 (92.9)	2.367	0.124

NRDS, neonatal respiratory distress syndrome; IVH, intraventricular hemorrhage; PVL, periventricular leukomalacia; BPD, bronchopulmonary dysplasia; NEC, necrotizing enterocolitis; ROP, retinopathy prematurity; LOS, late-onset neonatal sepsis; PH, pulmonary hemorrhage; PS, pulmonary surfactant.

Further subgroup analyses were conducted to determine the differences in morbidity between the two groups. Among the 646 VPIs with a gestational age of 28–31^+6^weeks, 317 were delivered vaginally and 329 were delivered via cesarean section. There were 134 VPIs with a gestational age <28 weeks, with 73 delivered vaginally and 61 delivered via cesarean section. Differences in the incidence rates of birth asphyxia, PH, NRDS, and the utilization of PS and invasive ventilation were predominantly observed in VPIs with a gestational age of 28–31^+6^ weeks. Additionally, the rate of delivery room resuscitation for VPIs delivered via cesarean section was higher than that for VPIs delivered vaginally at all gestational ages. There were no statistically significant differences between the two groups in different gestational age to the incidence rates of IVH, severe IVH, PVL, BPD, NEC, NEC surgery, ROP, severe ROP, LOS, or non-invasive ventilation support (Table 3).

**Table 3:** The morbidities based on different gestational age after PSM (%).

Morbidities	28 ~ 31^+6^w					<28w				
	Vaginal delivery (317)	Cesarean delivery (329)	Total (646)	χ^2^/Z	*p*	Vaginal delivery (73)	Cesarean delivery (61)	Total (134)	χ^2^//Z	*p*
Birth asphyxia	110 (34.7)	152 (46.2)	262 (40.6)	8.857	0.003	55 (75.3)	52 (85.2)	107 (79.9)	2.026	0.155
Delivery room resuscitation	204 (64.4)	266 (80.9)	470 (72.8)	22.168	<0.001	62 (84.9)	59 (96.7)	121 (90.3)	5.273	0.022
NRDS	193 (60.9)	252 (76.6)	445 (68.9)	18.596	<0.001	64 (87.7)	58 (95.1)	122 (91.0)	2.238	0.135
PS	172 (54.3)	225 (68.4)	397 (61.5)	13.608	<0.001	59 (80.8)	51 (83.6)	110 (82.1)	0.175	0.675
PH	14 (4.4)	39 (11.9)	53 (8.2)	11.859	<0.001	14 (19.2)	17 (27.9)	31 (23.1)	1.411	0.235
Invasive ventilation	83 (26.2)	145 (44.1)	228 (35.3)	22.625	<0.001	60 (82.2)	57 (93.4)	117 (87.3)	3.797	0.051
IVH	97 (30.6)	89 (27.1)	186 (28.8)	0.991	0.319	29 (39.7)	31 (50.8)	60 (44.8)	1.654	0.198
Severe IVH	18 (5.7)	26 (7.9)	44 (6.8)	1.259	0.262	12 (16.4)	12 (19.7)	24 (17.9)	0.236	0.627
PVL	6 (1.9)	9 (2.7)	15 (2.3)	0.506	0.477	2 (2.7)	4 (6.6)	6 (4.5)	–	0.411
BPD	80 (25.2)	81 (24.6)	161(24.9)	0.033	0.856	42(57.5)	38(62.3)	80(59.7)	0.313	0.576
NEC	21 (6.6)	20 (6.1)	41 (6.3)	0.081	0.776	2 (2.7)	5 (8.2)	7 (5.2)	–	0.245
NEC surgery	5 (1.6)	6 (1.8)	11 (1.7)	0.134	0.714	2 (2.7)	5 (8.2)	7 (5.2)	–	0.245
ROP	80 (25.2)	93 (28.3)	173 (26.8)	0.756	0.384	41 (56.2)	29 (47.5)	70 (52.2)	0.990	0.320
Severe ROP	1 (0.3)	3 (0.9)	4 (0.6)	-	0.624	5 (6.8)	2 (3.3)	7 (5.2)	–	0.454
LOS	31 (9.8)	27 (8.2)	58(9.0)	0.488	0.485	6 (8.2)	6 (9.8)	12 (9.0)	0.107	0.744
non-invasive ventilation	297 (93.7)	314 (95.4)	611 (94.6)	0.965	0.326	60 (82.2)	54 (88.5)	114 (85.1)	1.050	0.306

NRDS, neonatal respiratory distress syndrome; IVH, intraventricular hemorrhage; PVL, periventricular leukomalacia; BPD, bronchopulmonary dysplasia; NEC, necrotizing enterocolitis; ROP, retinopathy prematurity; LOS, ate-onset neonatal sepsis; PH, pulmonary hemorrhage; PS, pulmonary surfactant.

### 3.3 Mortality analysis

The number of deaths among VPIs after PSM was 69. Mortality rates were 10.3% (40/390) and 7.4% (29/390) in the vaginal and cesarean delivery groups, respectively. There were no differences in mortality rates between these two groups. The mortality rate of VPIs in the vaginal delivery group within 3 days after birth was 7.7% (30/390), which was significantly higher than the 3.3% (13/390) observed in the cesarean delivery group (*p* < 0.05). There were no significant differences in mortality rates between the two groups for VPIs with birth weights <1,000 g and 1,000–1,499 g. However, when analyzing gestational age, the mortality rate within three-day of VPIs in the vaginal delivery group was 50.0% (15/30) for those born at <28 weeks, which was higher than that in the cesarean delivery group (15.4%, 2/13, *p* < 0.05). Logistic regression analysis of three-day mortality among VPIs demonstrated that cesarean delivery was associated with a significantly lower mortality risk in VPIs with a gestational age of <28 weeks, with an adjusted odds ratio of only 2.6% (Table 4). The leading causes of death in the neonates born via vaginal delivery were NRDS (17/40, 42.5%), EOS (8/40, 20%), and severe pneumonia (4/40, 10%). The leading causes of death among the neonates born via cesarean section were NRDS (10/29, 34.5%), EOS (9/29, 31.0%), and NEC (3/29, 10.3%) (Table 5).

**Table 4:** Logistic regression analysis of mortality in VPIs within 3 days after birth.

Covariates	B	Standard Error	Wald	Sig.	Exp(B)
GA < 28 W	−3.657	1.254	8.503	0.004	0.026
BW < 1,000 g	1.860	0.997	3.483	0.062	6.422
Multiple	1.863	0.953	3.826	0.050	0.446
Constant	−1.820	0.807	5.092	0.024	0.162

BW, birth weight; GA, gestational age.

**Table 5:** The analysis of cause of mortality (%).

Mortality analysis	Vaginal delivery	Cesarean delivery	Total mortality	χ^2^/Z	*p*
Mortality	40/390 (10.3)	29/390 (7.4)	69/780 (8.8)	1.924	0.165
Mortality rate ≤3 days	30/390 (7.7)	13/390 (3.3)	43/780 (5.5)	7.113	0.008
BW < 1,000 g	16/30 (53.3)	9/13 (69.2)	25/43 (58.1)	0.248	0.619
GA < 28w	15/30 (50.0)	2/13 (15.4)	17/43 (39.5)	4.546	0.033
Multiple	16/30 (53.3)	8/13 (61.5)	24/43 (55.8)	0.942	0.332
Leading causes of death	NRDS (17/40, 42.5%) EOS (8/40, 20%) Severe pneumonia (4/40, 10%)	NRDS (10/29, 34.5%) EOS (9/29, 31.0%) NEC (3/29, 10.3%)			

BW, birth weight; GA, gestational age; NRDS, neonatal respiratory distress syndrome; EOS, early-onset neonatal sepsis; NEC, necrotizing enterocolitis.

## 4 Discussion

Considering the various morbidities that VPIs may encounter postnatally, a comprehensive analysis of the impact of cesarean versus vaginal delivery on their neurological and respiratory systems, as well as mortality, is essential for informed clinical decision-making.

The results of this study indicate that the incidence rates of early respiratory system diseases in VPIs delivered vaginally are lower compared with that in those delivered via cesarean section, including birth asphyxia, NRDS, and PH. Specifically, the incidence rates of birth asphyxia in cesarean delivery group is higher than in those vaginal delivery group, which is consistent with most current studies. Coviello et al. (12) reported that among pregnant women with preeclampsia, the incidence rates of birth asphyxia in preterm infants delivered via cesarean section were higher than those delivered vaginally. Similarly, Levin et al. (13) found a high incidence of birth asphyxia in premature infants delivered via cesarean section at <34 weeks of gestation, accompanied by prolonged hospital stays. This trend may be attributed to the fact that cesarean deliveries are often performed in cases of fetal distress or maternal emergencies, where the fetus may already be experiencing distress in utero. Furthermore, anesthesia may also adversely affect the respiratory system of VPIs.

Additionally, the results of this study showed that infants in the cesarean delivery group exhibited higher rates of resuscitation in the delivery room, which was associated with a higher incidence of birth asphyxia. Timely and effective resuscitation plays a crucial role in reducing the adverse outcomes of VPIs and enhancing survival rates. In contrast to vaginal delivery, cesarean delivery lacks vaginal squeezing, resulting in a greater volume of residual pulmonary fluid, a lower efficiency of fluid clearance, and reduced PS secretion. These factors contribute to the increased incidence of NRDS, as confirmed by multiple large-scale studies (14–16). Moreover, the occurrence of NRDS is closely associated with the administration of ACS. A complete course of ACS administered to mothers prior to delivery can reduce the incidence of NRDS. However, due to missing ACS data for 89 VPIs in this study, the PSM analysis of ACS administration was not feasible. Among the mothers, 58.3% (403/691) received a complete course of ACS before delivery, with no significant difference in ACS administration between the two groups (187/338 vs. 216/353, *p* > 0.05). The occurrence of PH is often associated with NRDS. According to a large-scale multicenter study conducted in China, the incidence of PH complicating NRDS in VPIs was 18.1%, and these infants usually presented with patent ductus arteriosus (PDA) and received PS therapy (17). PH is believed to arise from a rapid decrease in lung pressure, which increases the left-to-right shunting through the PDA, resulting in a rapid increase in pulmonary blood flow. The results of this study showed that the incidence rates of PH and PS use were higher in the cesarean delivery group than those in the vaginal delivery group. Furthermore, a high incidence of severe NRDS or PH in infants indicated the need for more invasive ventilation support. This may be related to a more severe deficiency of PS in premature infants delivered via cesarean section, with these differences being more pronounced in VPIs gestationally aged 28–31^+6^ weeks. Therefore, the management of ACS before cesarean delivery and high-quality resuscitation in the delivery room after birth are particularly important for the respiratory system management of VPIs.

Previous studies have suggested that vaginal delivery may increase the risk of IVH due to vaginal compression; however, most current research indicates that neither delivery modes increases the risk of severe IVH nor affects the long-term neurological outcomes (6, 18), which is consistent with this study. Owing to their immature intestinal development, preterm infants are at a high risk of developing NEC, characterized by severe intestinal inflammation and necrosis, which are the most common gastrointestinal emergencies among neonates. Dysbiosis of the intestinal flora is associated with increased susceptibility to NEC in this population (19). A high proportion of Firmicutes and low proportion of Bacteroidetes were found in infants undergoing cesarean delivery, which may be a potential factor in the development of NEC (19, 20). However, current research suggests that cesarean delivery does not increase the incidence of NEC (6, 21), which is consistent with the results of this study. However, the effects of intestinal flora dysbiosis in premature infants require further study. Additionally, Li et al. (21) compared the morbidities of 242 very low birth weight infants born via different delivery modes and concluded that cesarean delivery did not increase the incidence rates of ROP, PVL, or LOS, which is consistent with the results of this study.

Currently, there is no consensus regarding the effect of the two modes of delivery on the mortality rate of VPIs. A meta-analysis of 19 observational clinical trials involving 16,042 preterm infants showed that, compared with vaginal delivery, cesarean delivery could improve the survival rate of preterm infants (OR = 0.62, 95%CI: 0.42–0.90, *p* < 0.05) (7). Furthermore, according to the American College of Obstetricians and Gynecologists, cesarean delivery should be considered for VPIs born at a gestational age of 23–24 weeks and recommended for VPIs born at a gestational age of 25 weeks (22). This study also analyzed the mortality between the cesarean and vaginal deliveries and showed that cesarean delivery can reduce the mortality rate within 3 days postpartum after PSM, including multiple births. Further logistic regression analysis demonstrated that the protective effect of cesarean delivery against early mortality was most pronounced in VPIs with a gestational age <28 weeks, with minimal or no significant benefit observed in more mature preterm infants. Therefore, cesarean delivery should be considered for VPIs at <28 weeks to reduce the mortality in the early stage. The outcomes of VPIs in multiple pregnancies are not influenced by the modes of delivery, which is consistent with most current studies (6, 18, 23). An analysis of the causes of death showed that NRDS and EOS were important causes of death in both delivery modes, whereas severe pneumonia was a common cause of death in the vaginal delivery group. This is consistent with the main causes of death reported in VPIs and very low birth weight infants (24–26).

The effect of different delivery modes on early respiratory diseases in VPIs is significant. VPIs delivered vaginally have lower incidences of asphyxia, need for delivery room resuscitation, NRDS, and PH, as well as lower usage rates of PS and invasive ventilation than those delivered via cesarean section. These differences mainly occurred in VPIs with a gestational age of 28–31^+6^ weeks. However, there were no significant differences between the two groups in the incidences of IVH, PVL, BPD, NEC, ROP, and LOS, and usage rate of noninvasive ventilation. In addition, there was no difference in the total mortality rate between the two groups. For VPIs with a gestational age of <28 weeks, those delivered via cesarean section had a lower mortality rate within 3 days after birth than those delivered vaginally.

## 5 Limitations

This was a retrospective study, therefore, the number of VPIs with a gestational age <28 weeks was relatively limited and a substantial amount of data was lost during PSM. Furthermore, there may be a certain degree of bias regarding the impact of emergency cesarean section on conditions, such as birth asphyxia, delivery room resuscitation, and NRDS in VPIs. Since 52.1% of the cesarean sections were emergency procedures, this may have biased the results. Further study with larger sample sizes are required to validate these findings.

## Data Availability

The original contributions presented in the study are included in the article/supplementary material, further inquiries can be directed to the corresponding author.
